# Role of clusters in exclusive breastfeeding practices in Tanzania: A secondary analysis study using demographic and health survey data (2015/2016)

**DOI:** 10.3389/fped.2022.939706

**Published:** 2022-10-03

**Authors:** Ola Farid Jahanpour, Elphas Luchemo Okango, Jim Todd, Henry Mwambi, Michael Johnson Mahande

**Affiliations:** ^1^Department of Epidemiology and Biostatistics, Institute of Public Health, Kilimanjaro Christian Medical University College (KCMUCo), Moshi, Tanzania; ^2^Department of Mathematical Sciences, Strathmore University, Nairobi, Kenya; ^3^Department of Population Health, London School of Hygiene and Tropical Medicine, London, United Kingdom; ^4^National Institute for Medical Research, Mwanza, Tanzania; ^5^School of Mathematics, Statistics and Computer Science, University of KwaZulu-Natal, Pietermaritzburg, South Africa

**Keywords:** breastfeeding, mixed models/multilevel models, Tanzania, demographic health survey, secondary analysis

## Abstract

**Background:**

While the benefits of exclusive breastfeeding are widely acknowledged, it continues to be a rare practice. Determinants of exclusive breastfeeding in Tanzania have been studied; however, the existence and contribution of regional variability to the practice have not been explored.

**Methods:**

Tanzania demographic and health survey data for 2015/2016 were used. Information on infants aged up to 6 months was abstracted. Exclusive breastfeeding was defined using a recall of feeding practices in the past 24 h. Enumeration areas and regions were treated as random effects. Models without random effects were compared with those that incorporated random effects using the Akaike information criterion. The determinants of exclusive breastfeeding were estimated using the generalized linear mixed model with enumeration areas nested within the region.

**Results:**

The generalized linear mixed model with an enumeration area nested within a region performed better than other models. The intra-cluster variability at region and enumeration area levels was 3.7 and 24.5%, respectively. The odds of practicing exclusive breastfeeding were lower for older and male infants, for mothers younger than 18, among mothers residing in urban areas, among those who were employed by a family member or someone else, those not assisted by a nurse/midwife, and those who were not counseled on exclusive breastfeeding within 2 days post-delivery. There was no statistical evidence of an association between exclusive breastfeeding practices and the frequency of listening to the radio and watching television. When mapping the proportion of exclusive breastfeeding, a variability of the practice is seen across regions.

**Conclusion:**

There is room to improve the proportion of those who practice exclusive breastfeeding in Tanzania. Beyond individual and setting factors, this analysis shows that a quarter of the variability in exclusive breastfeeding practices is at the community level. Further studies may explore the causes of variabilities in regional and enumeration area and how it operates. Interventions to protect, promote, and support exclusive breastfeeding in Tanzania may target the environment that shapes the attitude toward exclusive breastfeeding in smaller geographical areas.

## Introduction

Exclusive breastfeeding (EBF) for the first 6 months of life improves the chances of survival for neonates and infants (1) by providing protection against multiple infections and reducing all-cause mortalities (2). Later in life, the benefits of exclusive breastfeeding to the child include reduced risks of developing obesity and developing non-communicable diseases, such as Type 2 diabetes mellitus (3). Exclusive breastfeeding has also been associated with better performance in intelligence tests (4). Despite its benefits, EBF is a rare practice. The latest estimates of infants who are exclusively breastfed at the global level and in Tanzania are below 90% (5, 6).

To protect, promote, and support exclusive breastfeeding, policies, strategies, and interventions are developed based on an understanding of the determinants of EBF (2). One conceptual model to describe the enabling environment for breastfeeding has grouped the determinants into three groups: individual, settings, and structural (2). Individual level determinants of EBF include infant-related factors, such as age (7, 8), sex (9–11), singleton or twin (9), and mother's attributes, such as age (9), level of education (7, 9, 12), marital status (12), number of children (10), area of residence (8, 10, 11), wealth index (9–11), and alcohol intake (13). Setting-related factors include the place of delivery (9, 10, 14), provision of counseling on EBF after delivery (13), paternal level of education (13), occupation (10), and the practices and experiences among others in the community (2).

The conceptual model referred to above describes structural factors as the social context that can shape the attitude toward EBF by creating an environment that is either supportive of or opposing EBF (2). The social context may be influenced by social trends, advertising, socio-cultural norms, and beliefs, which, while reaching the whole population, affect differently the different segments of the population (2). And thus, EBF practices can truly be a local phenomenon (15) that may be determined by factors beyond an individual's socio-demographic attributes (16). For example, Yalcin et al. established that approximately 12.2% of the differences in the proportion of EBF among sub-Saharan countries were due to country-level differences (9). There is limited literature on the influence of the social context on EBF practices in Tanzania (17). Understanding the existence of this social context and its influence on breastfeeding practices is crucial to guide interventions.

Using classical statistical methods, it is challenging to evaluate the effect of the socio-context unless the socio-context is put as a variable and treated as one of the exposures. Advanced statistical methods, such as mixed models, may be used to evaluate the influence of social context on EBF practices and quantify it (18). The model evaluates the influence of a social context in two ways. One, the model accounts for a correlation between subjects, if any, when establishing the estimates of the outcome of interest by accommodating the hierarchy in the data through the use of random effects. Two, the model estimates the contribution of the variability in the outcome of interest that results from the effect of the social context through establishing the degree of between and within correlations in clusters. Different from classical approaches, mixed models account for the independence of observations, which is expected in data such as those collected during Demographic and Health Surveys (DHS). In DHS, participants are clustered within an enumeration area, which is nested in a region. With regards to EBF practice, the women in an enumeration area may be more similar compared to women from other enumeration areas and may share concerns and information about EBF. Ignoring this association may lead to underestimating the standard error which in effect increases the chance of type I error (19). Using the Tanzania Demographic and Health Survey data (TDHS) 2015/2016, this study uses generalized linear mixed models to estimate the determinants of EBF accounting for the correlation in clusters, at the enumeration area and regional level, and estimates the degree of the variability in the proportion of EBF that is attributed by this correlation.

## Methods

### Data source

Data used in this study was the Tanzania 2015/2016 DHS data. Details on DHS have been described by Croft et al. (20). At the country level, a sampling frame is usually obtained. To minimize sampling errors, the country is stratified by geographic region and by urban/rural areas within each region, followed by a two-stage sampling to select a household to be surveyed. The first sampling is to select a primary sampling unit (PSU) and then select a household. PSUs are survey clusters that are usually based on census enumeration areas (EAs). A probability proportion to size is employed in each stratum to select the PSU. For each selected PSU, a complete household listing is done. This is then followed by selecting a fixed number of households to be surveyed using equal probability systematic sampling. Because of this, each participant will have a different probability of participating in the survey. To adjust for differences in the probability of selection, sampling weights are employed during the analysis.

**Figure 1:**
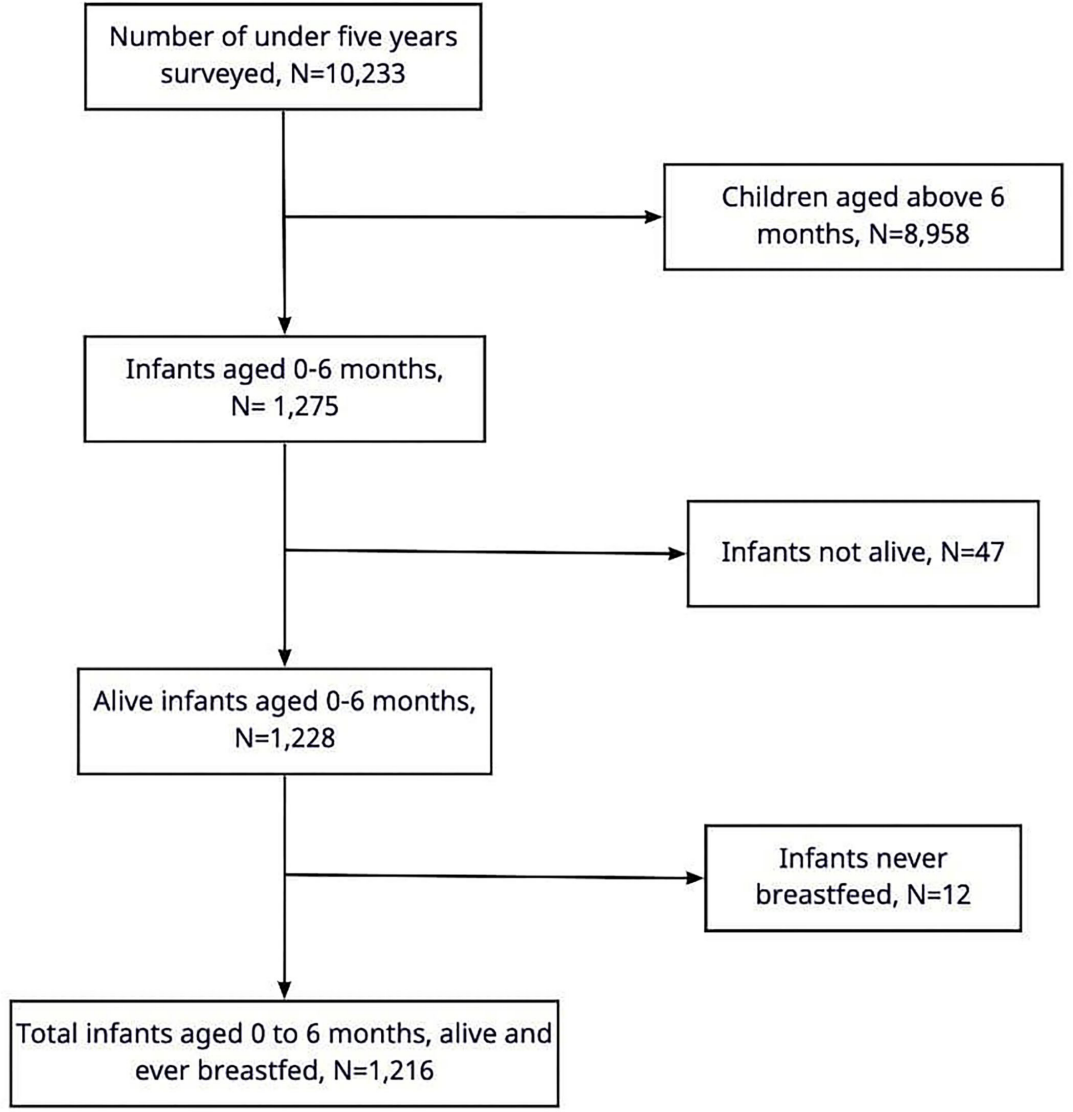
Participants' flow.

At a household, women and men of reproductive age who are residents or who slept in a household the night before the survey are eligible participants. In its data collection, the survey uses four questionnaires: household, women, men, and biomarkers. The women's questionnaire asks questions about women and also those related to their children. The TDHS 2015/2016 was conducted in the whole country where it was divided into 59 sampling strata and 608 EAs, interviewing about 13,000 women aged 15–49 and 3,200 men aged 15–49. For this study, information on infants aged 0–6 months was extracted. As shown in Figure 1, the 2015/2016 TDHS had surveyed 10,233 children under 5 years, of this, 8,958 were dropped as they were above the age of 6 months. Of those who were aged 0–6 months, 47 were dropped as they were not alive at the time of data collection and 12 were dropped as they were never breastfed. The information from 1,216 infants was analyzed in this study. Since the study used information on feeding in the past 24 h, infants who died before the age of 6 months were excluded.

### Data analysis

#### Measurement of variables

The outcome of interest was EBF at 0–6 months based on 24 h recall, which is the method used in DHS. Infants who were provided with only breast milk and prescribed medications were categorized as those who were exclusively breastfed. In the data set, 16 infants were grouped as currently not breastfeeding, and who had no information given on the variables, indicating whether they had been given anything other than breast milk or whether they ate any solid, semi-solid, or soft food the day before, were counted as not exclusively breastfed. There were two (2) infants who were still breastfeeding and had missing information on eating solid, semi-solid, or soft foods the day before but were not given anything other than breast milk, they were therefore counted as practicing exclusive breastfeeding.

Covariate selection for the model was based on previous literature reports (7–14, 17, 21–23), availability of data, and a two-stage variable selection approach. Through this process, the following explanatory variables were selected: infant's and mother's socio-demographic characteristics, setting, and structural factors. For infants, the variables included sex and age in months. Mothers' characteristics included: age in years, literacy, marital status, area of residence, wealth index, work history, and frequency of listening to the radio and watching television (TV). Mother's variables were categorized as follows: age in years: categorized into < 18, 18–24, and equal to or above 25; literacy: cannot read at all, able to read only parts of sentences, and ability to read the whole sentence; marital status, which was put into groups, those who were living with a partner during the interview and those that were not; area of residence: rural and urban; wealth index was categorized into five (5) categories: poorest to richest; work history: categorized into working for a family member or someone else, self-employed, and not working; frequency of listening to the radio and television, which were both categorized as not at all, less than once a week, and at least once a week. Two variables were included under the setting factors: assistance by a midwife/nurse and counseling on breastfeeding during the first two (2) days post-delivery by a healthcare provider, which were categorized into yes and no. Regions of residence and enumeration areas were included and were used in estimating the structural factors. TDHS 2015/2016 does not have any data on the HIV knowledge and status of the participants, and therefore, HIV status, although a significant contributor to EBF practices, was not used in the model.

#### Data analysis

Unweighted and weighted frequency, proportion, and 95% confidence interval were used to summarize the covariates. Using the QGIS software, a map of the proportion of EBF at the regional level was developed using the weighted proportion of EBF at the regional level.

The two-stage variable selection approach was first carried out using the Chi-square test and then using the backward selection. The Chi-square test was used to determine variables that significantly made a difference between the group, which exclusively breastfed at 0–6 months, and those who did not. This then assisted in guiding a decision on which variables to include in the backward selection. Based on the Chi-square test, the following covariates were selected to be used for the second stage selection: infant's age and sex, mother's age, literacy, marital status, area of residence, wealth index, work history, assistance by midwife/nursed, counseling on breastfeeding, and frequency of listening to the radio and television. As shown in Table 1, sex of the infant, mother's age, current marital status, wealth index, mother's residence, frequency of listening to the radio, and frequency of watching television were not statistically significant (*p* > 0.05), they were, however, included in the final model as they have been reported as key determinants in previous studies. And the frequency of listening to radio or TV was included in the final model as our team wanted to explore their contribution to influence breastfeeding practices.

**Table 1:** Background characteristics of the participants (*N* = 1,216).

	**Overall total (** ***a*** **)**		**Total EBF (** ***b*** **)**		**Yes EBF (** ***b*** **)**		***p*-value**
	***n***	**%**	**%**	**95% CI**	**%**	**95% CI**	
	1,216				57.7	[54.1, 61.2]	
**Sex of an infant**							0.48
Male	598	49.2	50.8	[47.6, 54.1]	56.6	[52.1, 61.0]	
Female	618	50.8	49.2	[45.9, 52.4]	58.8	[53.8, 63.7]	
**Infant's age (months)**							< 0.001
0	196	16.1	16.1	[13.9, 18.6]	82.8	[76.4, 87.8]	
1	188	15.5	15.6	[13.2, 18.2]	75.7	[67.3, 82.5]	
2	175	14.4	15.6	[13.3, 18.2]	74.3	[66.0, 81.2]	
3	160	13.2	13.4	[11.4, 15.7]	64.1	[55.1, 72.2]	
4	178	14.6	13.5	[11.4, 15.9]	52.2	[43.6, 60.5]	
5	125	10.3	10.2	[8.4, 12.5]	30.9	[22.3, 41.2]	
6	194	16	15.6	[13.4, 18.1]	14	[8.9, 21.3]	
**Mother's age (years)**							0.063
< 18	62	5.1	5.9	[4.5, 7.7]	41.7	[28.4, 56.3]	
18–24	485	39.9	41.2	[37.6, 44.9]	59.8	[54.2, 65.2]	
>25	669	55	52.9	[49.5, 56.3]	57.8	[53.1, 62.4]	
**Literacy**							0.05
Cannot read at all	343	28.2	28.5	[25.5, 31.6]	51	[44.8, 57.2]	
Able to read only parts of sentence	64	5.3	5.5	[4.0, 7.4]	56.8	[40.9, 71.5]	
Able to read whole sentence	809	66.5	66.1	[62.6, 69.4]	60.6	[56.4, 64.7]	
**Current marital status**							0.502
Never in union/widowed/divorced/no longer living together	169	13.9	15.5	[13.2, 18.1]	60.4	[51.3, 68.9]	
Married/living with partner	1,047	86.1	84.5	[81.9, 86.8]	57.2	[53.4, 60.9]	
**Wealth index**							0.415
Poorest	298	24.5	25.8	[22.2, 29.7]	52.5	[46.5, 58.4]	
Poorer	254	20.9	21.8	[18.9, 24.9]	61.5	[54.2, 68.4]	
Middle	217	17.8	17.4	[14.9, 20.1]	58.6	[50.4, 66.3]	
Richer	249	20.5	19.7	[16.8, 23.0]	59.4	[51.2, 67.0]	
Richest	198	16.3	15.3	[12.9, 18.1]	57.7	[49.1, 65.8]	
**Mother's residence**							0.284
Urban	287	23.6	27.4	[23.8, 31.3]	54.3	[46.6, 61.8]	
Rural	929	76.4	72.6	[68.7, 76.2]	59	[54.9, 62.9]	
**Who respondent works for**							0.014
For family member/someone else	442	36.3	37.4	[33.6, 41.2]	50.9	[45.0, 56.7]	
Self-employed	497	40.9	39.4	[36.0, 42.9]	61.8	[56.4, 67.0]	
Not working	277	22.8	23.2	[19.2, 27.8]	61.5	[53.9, 68.5]	
**Assistance: nurse/ midwife**							0.014
No	506	41.6	40.9	[37.0, 45.0]	52.8	[47.5, 58.1]	
Yes	710	58.4	59.1	[55.0, 63.0]	61	[56.6, 65.3]	
**During first 2 days health provider: counsel on breastfeeding**							0.028
No	824	67.8	65.1	[61.4, 68.6]	54.8	[50.6, 59.0]	
Yes	392	32.2	34.9	[31.4, 38.6]	63	[56.8, 68.8]	
**Frequency of listening to radio**							0.117
Not at all	312	25.7	25.8	[22.4, 29.5]	58.9	[52.6, 65.0]	
Less than once a week	436	35.9	36.8	[33.5, 40.3]	53.3	[47.3, 59.2]	
At least once a week	468	38.5	37.4	[34.0, 40.9]	61.1	[55.6, 66.3]	
**Frequency of watching television**							0.918
Not at all	700	57.6	59.2	[55.2, 63.1]	58	[53.4, 62.4]	
Less than once a week	282	23.2	22.6	[19.7, 25.8]	56.4	[49.3, 63.3]	
At least once a week	234	19.2	18.2	[15.5, 21.2]	58.2	[50.4, 65.6]	

*A*, unweighted cases; *b*, weighted.

The backward selection at a *p*-value of < 0.2 was used to identify the covariates to include in the model. This *p*-value was set high to provide room for a liberal criterion and be able to rule out confounding factors more effectively (19). The variables selected were: age and sex of an infant, mother's age, area of residence, literacy, wealth index, work history, assisted by a nurse/midwife, counseled on EBF, frequency of listening to the radio, and watching television. Akaike information criteria (AIC) were used in model selection, whereby a model with a smaller score was selected. The Melogit package in STATA version 16 was used to explore factors associated with EBF accounting for the hierarchical structure of the data using generalized linear mixed models. Three models were compared:

Model 1 (standard logistic model): *h*(*p*_*ij*_) = β_0_+ **βX**

Model 2 (random effects model): *h*(*p*_*ij*_) = β_0_ + **βX** + μ_*i*_

Model 3 (nested random effects model): *h*(*p*_*ijk*_) = *Xβ*_*k*|*i*_ + **X*****β***_*i*_ + μ_*k*|*i*_ + μ_*i*_

where

*h*(*p*_*ij*_) and *h*(*p*_*ijk*_) are logit link functions describing the logs of odds of child *j* in region *i* and enumeration area *k*,

β′*s* are the regression coefficients,

*X*′*s* are the covariates,

μ_*i*_ is the region-specific random effects,

μ_*k*|*i*_ is the random effects capturing the variation due to different enumeration areas *k* within a common region *i*,

*Xβ*_*k*|*i*_ captures how enumeration areas affect the slope of *X* relationship given common region,

*Xβ*_*i*_ captures how region affects the slope of *X*.

Interaction between residence and who hired the respondent (self-employed, hired by a family member or someone else, or not employed) and between sex of an infant and residence (rural or urban) were tested. The interaction between the residence and who hired the respondent was statistically significant but did not improve the models' performance and therefore was not included in the final model. The third model was the final model. Overall characteristics of the sample are given as unweighted case numbers and percentages, whereas overall EBF prevalence and EBF practices by different independent variables are reported as weighted percentages. A *p*-value of < 0.05 was considered statistically significant.

## Results

### Background characteristics of the participants

There was a total of 1,216 infants from 30 regions and 486 enumeration areas, the remaining 122 (20%) enumeration areas did not have an infant under the age of 6 months. There was an average of 40 infants within a region with a minimum of three (3) and a maximum of 100. A total of 57.7% (95% CI (confidence interval) (54.1, 61.2%) practiced EBF (Table 1).

**Figure 2:**
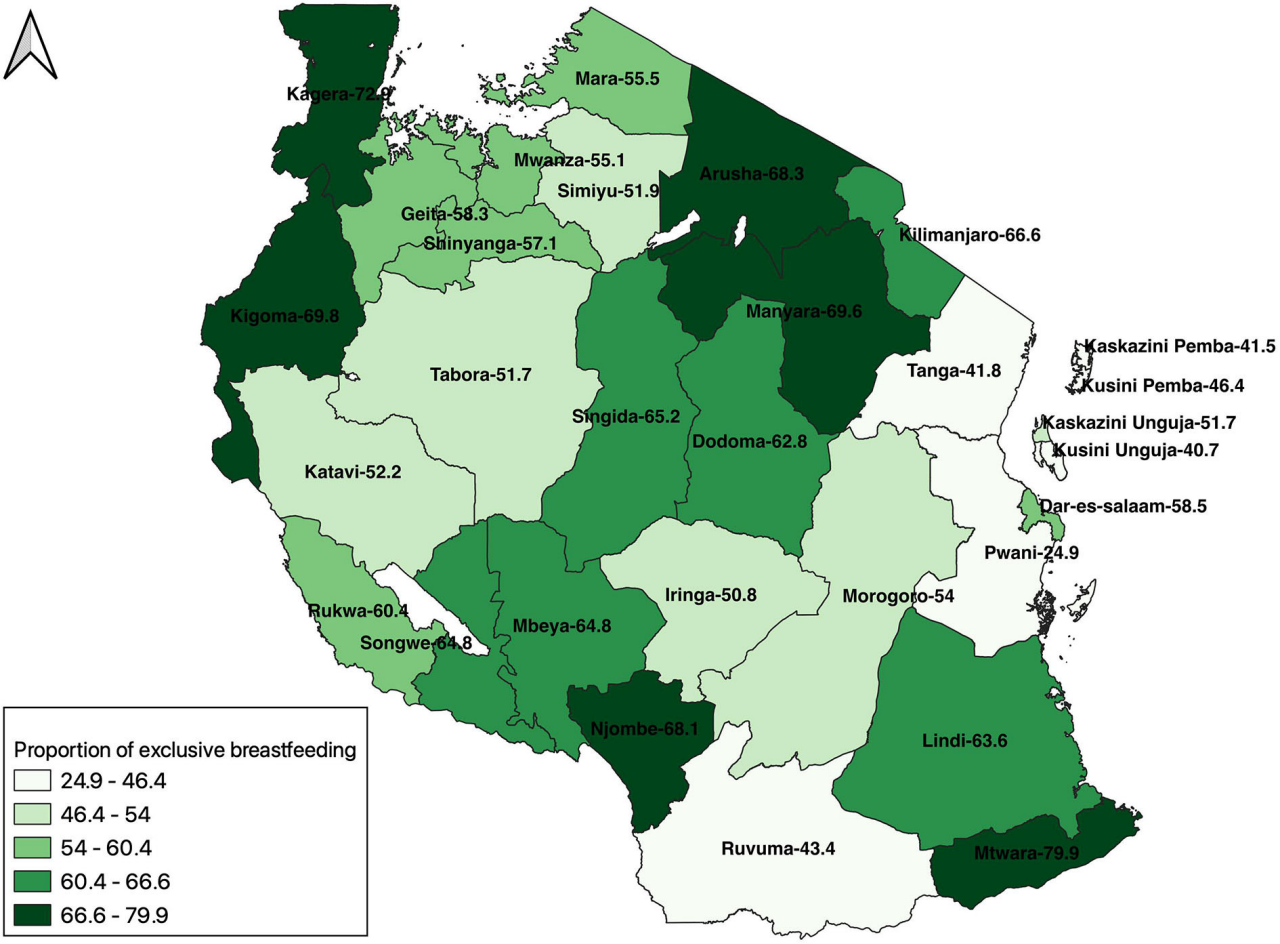
A map of the proportion of exclusive breastfeeding in Tanzania, 2015/2016.

A map of the proportion of exclusive breastfeeding in Tanzania shows the variability of the practice across the country. Generally, the coastal region of Tanzania mainland and those in Zanzibar have the lowest proportion. Ruvuma, which is not at the coast, has a proportion of 43.4%, which is lower than the national average. Mtwara, although a coastal region, has the highest proportion in the country. Other regions with the highest proportion of EBF are scattered in the country and found in Kagera (North-west), Kigoma (West), Arusha (North), Manyara (North), and Njombe (South) (Figure 2).

As shown in Table 1, there was an almost equal number of male and female infants (49.2 vs. 50.8%). The majority of women were aged 25 years and older (55%), while 5.1% were < 18, and 39.9% were aged 18–24 years. More than half of the participants (66.5%) could read, 86.1% were married or living with a partner, and 76.4% were from rural areas. About a quarter of the participants (22.8%) were not working, 36.3% were working for a family member or someone else and 40.9% were self-employed. More than half (58.4%) were assisted by a nurse or midwife during delivery and 32.2% were counseled on breastfeeding during the first 2 days post-delivery. Participants listened to the radio more than watching television. A quarter of the women (25.7%) did not listen to the radio at all, 35.9% listened less than once a week, and 38.5% at least once a week. More than half of the women (57.6%) did not watch television at all, 23.2% watched less than once a week, and 19.2% at least once a week (Table 1).

Also shown in Table 1 and as described in the data analysis section, at bivariate analysis, only the infant's age had a statistically significant contribution to EBF practices (*p* < 0.001), while the infant's sex did not (*p* = 0.48). Regarding the mother's factors, those that were statistically significant were working history (*p* = 0.014), assistance by a midwife/nurse (*p* = 0.014), and being counseled on EBF post-delivery (*p* = 0.028), while those that were not were the age of the mother (*p* = 0.063), marital status (*p* = 0.502), wealth index (*p* = 0.415), area of residence (*p* = 0.284), frequency of listening to a radio (*p* = 0.117), and watching TV (*p* = 0.918).

### Factors associated with exclusive breastfeeding with enumeration areas (clusters) nested in regions as random factors

Table 2 shows the results from the final model with crude and adjusted odds ratio. The final model was developed by adjusting for infant's age and sex, mother's age, type of residence, literacy level, wealth index, mother's working status, assistance by nurse/midwife, counseling on breastfeeding within the first 2 days, frequency of listening to the radio, and frequency of watching television. When adjusting for all these factors, infant's age and sex, mother's age, type of residence, mother's working status, assistance by nurse/midwife, and counseled on breastfeeding during the first 2 days are associated with EBF; however, literacy level, wealth index, frequency of listening to radio and frequency of watching television are not associated with EBF (Table 2).

**Table 2:** Factors associated with exclusive breastfeeding with enumeration areas nested in regions as random factors.

**Fixed factors**	**Crude odds ratio**	***p*-value**	Adjusted odds ratio^*^	***p*-value**
**Current infant's age (months)**				
0	1		1	
1	0.71 (0.390, 1.284)	0.256	0.67 (0.360, 1.239)	0.2
2	0.55 (0.304, 0.990)	0.046	0.48 (0.257, 0.882)	0.018
3	0.30 (0.165, 0.541)	< 0.001	0.25 (0.136, 0.469)	< 0.001
4	0.16 (0.090, 0.297)	< 0.001	0.14 (0.076, 0.266)	< 0.001
5	0.06 (0.032, 0.119)	< 0.001	0.05 (0.027, 0.108)	< 0.001
6	0.02 (0.009, 0.035)	< 0.001	0.01 (0.006, 0.026)	< 0.001
**Sex of an infant**				
Male	1		1	
Female	1.17 (0.891, 1.542)	0.256	1.58 (1.136, 2.200)	0.007
**Mother's age (year)**				
< 18	1		1	
18–24	2.64 (1.437, 4.867)	0.002	2.69 (1.297, 5.575)	0.008
25+	2.21 (1.222, 3.994)	0.009	2.98 (1.451, 6.122)	0.003
**Type of residence**				
Urban	1		1	
Rural	1.34 (0.908, 1.971)	0.141	1.94 (1.100, 3.434)	0.022
**Literacy**				
Cannot read at all	1		1	
Able to read only parts of sentence	1.16 (0.613, 2.194)	0.649	1.16 (0.541, 2.503)	0.699
Able to read whole sentence	1.42 (1.043, 1.937)	0.026	1.18 (0.795, 1.742)	0.416
**Wealth index**				
Poorest	1		1	
Poorer	1.61 (1.062, 2.442)	0.025	2.04 (1.233, 3.375)	0.006
Middle	1.34 (0.853, 2.092)	0.205	1.33 (0.777, 2.288)	0.297
Richer	1.48 (0.945, 2.332)	0.087	1.31 (0.714, 2.390)	0.386
Richest	1.30 (0.779, 2.178)	0.313	1.15 (0.493, 2.690)	0.745
**Mother's working status**				
Working for family member/Someone else	1		1	
Self-employed	1.58 (1.145, 2.184)	0.005	1.98 (1.343, 2.924)	0.001
Not working	1.56 (1.059, 2.294)	0.024	1.88 (1.179, 3.011)	0.008
**Assistance by nurse/midwife**				
No	1		1	
Yes	1.54 (1.144, 2.072)	0.004	1.63 (1.101, 2.404)	0.015
**During first 2 days health provider: counsel on breastfeeding**				
No	1		1	
Yes	1.42 (1.045, 1.942)	0.025	1.49 (1.002, 2.227)	0.049
**Frequency of listening to radio**				
Not at all	1		1	
Less than once a week	0.78 (0.544, 1.122)	0.181	0.97 (0.621, 1.526)	0.906
Almost every day	1.08 (0.754, 1.549)	0.671	1.21 (0.756, 1.941)	0.426
**Frequency of watching television**				
Not at all	1		1	
Less than once a week	0.88 (0.621, 1.256)	0.489	0.79 (0.506, 1.219)	0.282
Almost every day	0.92 (0.617, 1.385)	0.703	1.37 (0.731, 2.565)	0.327
**Random effects**				
Variance (enumeration areas)			1.067564	
Variance (regions)			0.1256284	
ICC (enumeration areas)			0.24515979	
ICC (regions)			0.0368128	
*p*-value			< 0.0001	

* Adjusted for infant's age and sex, mother's age, residence, literacy, wealth index, working status, assistance by nurse/midwife, counseled on breastfeeding within first 2 days, frequency of listening to radio and frequency of watching television.

Exclusive breastfeeding is less likely practiced as an infant ages and the difference starts to be statistically significant at the age of 2 months. Compared with male infants, female infants were more likely to be exclusively breastfed (AOR=1.58; CI 95% 1.14–2.2; *p* = 0.007). The odds of practicing EBF increased by about 2-folds for women who were above 18 years old compared to those who were younger than 18. Women in rural areas were more likely to practice EBF than those in urban areas (AOR=1.94; CI 95% 1.1–3.4; *p* = 0.022). Mothers who were self-employed (AOR = 1.9; CI 95% 1.2–3.0; *p* = 0.008) or not working (AOR=1.98; CI 95% 1.3–2.9; *p* = 0.001) were more likely to practice EBF compared to those employed by a family member or someone else. Women who were assisted by a nurse/midwife were more likely to practice EBF than those who were not (AOR = 1.63; CI 95% 1.1–2.4; *p* = 0.015). Women who were counseled on breastfeeding within 2 days post-delivery were more likely to practice EBF (AOR = 1.49, CI 95% 1.002–2.2; *p* = 0.049). While the frequency of listening to the radio and of watching television had no statistical evidence of increasing the odds of practicing EBF, those who listened to the radio every day and those who watched television every day were more likely to practice EBF compared with those who did not at all (Table 2).

Looking at the random effect estimates, in a model without covariates (empty model), the intra-cluster correlation was 23.7% with enumeration areas as the only random effect and 2% with regions as the only random effect. In a model without the covariates with enumeration areas nested in the region, the intra-cluster correlation for regions was 1.6 and 23.1% for enumeration areas. In the final model, there was a justification for including enumeration areas nested in regions as random effects in the model (*p*-value < 0.0001). In this model, the proportion of the variance explained by the between regions' variations was 3.7%, and that by enumeration areas was 24.5% (Table 2).

## Discussion

In this study, the determinants of exclusive breastfeeding in Tanzania were established using generalized linear mixed models with enumeration areas nested in regions as random factors. The study shows that even when accounting for random factors, individual and setting factors influence EBF practices in Tanzania. The current study shows that it is plausible to consider random effects of regions and enumeration areas when analyzing determinants of breastfeeding in Tanzania using hierarchical data, such as DHS data. The study also shows that about a quarter of the variability in EBF practices is contributed by structural factors in the enumeration area.

Regarding the individual determinants, this study shows that both infant's and mother's factors contribute to EBF practices. Looking at infant's factors, the study shows that there is a decrease in EBF as an infant gets older. This trend has been reported in other studies in Tanzania (7, 24) and in other countries (8). This decrease may be contributed by mothers resuming work and mothers thinking that breastmilk is not sufficient for an aging infant. Understanding the age at which there is a general trend to stop EBF may be used to guide interventions on the time to focus so as to promote EBF practices and ensure it is done when the infant is 6 months old as recommended. This study also shows that the sex of the infant influences EBF practices and that a female infant is more likely to be exclusively breastfed. This finding is similar to studies done in other part of Africa (9, 11) and different from a study done in a different continent where sex did not influence EBF practices (10). The perceived hunger of the child influences the mother's feeding practices (2) and mothers may assume that a male infant demands more food and not be satisfied by just breastmilk. Understanding the social context and the perception of mothers and the community on the perceived hunger of their children may help in raising an awareness that male infants too need to be exclusively breastfed.

Regarding the mother's factors, this study shows that an increase in the mother's age is associated with an increased odds of practicing EBF. This could be because as a woman ages her autonomy increases and also this woman might have been exposed to multiple counseling sessions that encourage EBF during previous pregnancies (16). Family planning that would help avoid pregnancies at a younger age may indirectly reduce the proportion of infants not exclusively breastfed. This study shows that women who were working for someone else were less likely to practice EBF. This could be due to the policy at work that does not support EBF practices (2) and also could be due to the high workload that is making it hard to practice EBF. Emphasis is to continue to be made to ensure that environment at work supports a mother's decision to practice EBF. And also, there is a need for social support to decrease the workload for a breastfeeding mother, so as to permit her to exclusively breastfeed.

Regarding the setting factors, mothers who were assisted by a nurse/midwife and those who were counseled on breastfeeding were more likely to practice EBF. A study done in a hospital setting in Tanzania found that socio-demographic characteristics of the mother did not influence EBF practices but counseling provided at the hospital was attributed to influence EBF practices (16). Counseling and education have been found to improve the proportion of EBF (25), especially when provided concurrently at the health facility and in the community. Tanzania should continue to provide counseling to promote EBF at health facilities and in the community and improve the quality of the counseling provided. In Tanzania, the role of residence in EBF practices has varied, some studies report that women in urban have done better than those in rural (21), while others reported no differences (24). Our study shows that those in rural had higher odds of practicing EBF than those in urban. Reasons for women in urban having higher odds of practicing EBF included delivering at Baby Friendly facilities and therefore exposed to an environment conducive to supporting and promoting EBF (21). This may be possible that delivering at a health facility (22) and being counseled on EBF post-delivery (13) have been reported to increase the odds of practicing EBF, and was also shown to be the case in our study. Further studies may be conducted to evaluate the role of a place of residence and its relationship with one's wealth quantiles and guide interventions.

Education plays a role in a mother's feeding practices (8, 24) as a way to provide a mother with the needed information and equip her with skills that would facilitate appropriate feeding practices. Our study looked at literacy level and found no statistical evidence of an association between literacy and EBF practices as this is perhaps due to the fact that EBF practices would be influenced by the current capacities of the woman in accessing and processing information rather that her education history. This then raises the question of how to enable a mother to have ongoing access to information on EBF particularly the most vulnerable ones who are less likely to practice EBF. These vulnerable women are most likely not assisted by a nurse/midwife during delivery and not counseled on EBF post-delivery; it is perhaps going to be challenging to reach them through interventions at health facilities. While not statistically significant, women who listened to the radio or watched television had higher odds of practicing EBF. The use of mass media has been found effective in improving the proportion of EBF (26) and Tanzania may consider using them extensively. The high proportion of the variability found in the enumeration areas (clusters) perhaps points to another way health information messages can be improved. Effective community mobilization would help spread positive messages and improve EBF coverage.

Variability of EBF practices per geographical area has also been reported in other studies (8, 10, 11, 15, 27). The current study has also quantified the variabilities in the proportion of EBF that results from regional and enumeration areas differences and shows that greater variability is in enumeration areas. This confirms that women in a closer geographical area tend to have similar breastfeeding practices and also shows that Tanzania is heterogeneous when it comes to feeding practices. This also shows that there could be cultural differences in communities, based on local information and practice, that are influencing EBF practices. As the march toward improving the proportion of women who practice EBF moves forward, studies may be designed to explore the pathway through which these variabilities operate in these smaller areas. Also, as we head toward the deadline of meeting the Sustainable Development Goals targets in 2030 and the Global Nutrition Target in 2025, interventions in these smaller geographical areas may be designed and implemented to ensure meeting the targets. Tanzania has adopted a decentralization policy with decision-making and implementation to improve the wellbeing of the citizens being made at the district level (few enumeration areas make a district), hence a structure to support the design and implementation of these interventions is in place (17) and may be used.

Being a cross-sectional study, this study cannot establish a causal effect of EBF practices. The use of the 24-h recall method to establish the proportion of EBF has been reported to lead to elevated estimates of EBF (28). However, this method is acceptable (29). DHS is vigorous in its sampling techniques and thus ensures a good representation of the whole country. Also, DHS uses wellformulated tools and trains its data collectors well. The use of a mixed model has accounted for correlation in the data, and thus improved the estimates of the determinants of EBF. Also, being a quantitative study, the exact contextual factors influencing EBF practices cannot be pinpointed. However, using generalized linear mixed models, this study has been able to establish that contextual factors exist and have been able to quantify their influence on EBF practices.

## Conclusion

The proportion of exclusive breastfeeding in Tanzania still needs to be improved. In Tanzania, there is significant variability in the proportion of exclusive breastfeeding contributed to regional and enumeration areas differences. While some of the individual determinants for exclusive breastfeeding are known, it might be challenging to reach the population that needs help the most to protect, promote, and support exclusive breastfeeding. Interventions to improve EBF may focus on changing the socio-cultural norms and general attitude toward the practice. These practices may be different for different geographical areas and thus specific geographical interventions may be designed. Further studies are needed to be conducted to explore the causes of the variability in these smaller geographical areas.

## Data availability statement

The author selected the following statement: Publicly available datasets were analyzed in this study. Data may be obtained at https://dhsprogram.com/ upon permission from Measure DHS.

## Author contributions

OJ requested the data and the research concept was developed. Data analysis was done by OJ under the guidance and support of EO, JT, HM, and MM. The first draft of the manuscript was developed by OJ, all authors read and approved the final manuscript.

## Funding

This study was supported through the DELTAS Africa Initiative SSACAB (Grant No. 107754/Z/15/Z). The DELTAS Africa Initiative is an independent funding scheme of the African Academy of Sciences (AAS) Alliance for Accelerating Excellence in Science in Africa (AESA) and is supported by the New Partnership for Africa's Development Planning and Coordinating Agency (NEPAD Agency) with funding from the Wellcome Trust (Grant No. 107754/Z/15/Z) and the UK government.

## Conflict of interest

The authors declare that the research was conducted in the absence of any commercial or financial relationships that could be construed as a potential conflict of interest.

## Publisher's note

All claims expressed in this article are solely those of the authors and do not necessarily represent those of their affiliated organizations, or those of the publisher, the editors and the reviewers. Any product that may be evaluated in this article, or claim that may be made by its manufacturer, is not guaranteed or endorsed by the publisher.

## References

[1] Victora CG Bahl R Barros AJD França GVA Horton S Krasevec J. Breastfeeding in the 21st century: epidemiology, mechanisms, and lifelong effect. Lancet. (2016) 387:475–90. 10.1016/S0140-6736(15)01024-7 26869575

[2] Rollins Ni. Bhandari Nita, Hajeebhoy Nemat, Horton Susan, Lutter Chessa MJ. Breastfeeding series group, why invest, and what it will take to improve breastfeeding practices? Lancet. (2016) 387:491–504. 10.1016/S0140-6736(15)01044-2 26869576

[3] Horta BL Loret de Mola C Victora CG. Long-term consequences of breastfeeding on cholesterol, obesity, systolic blood pressure and type 2 diabetes: a systematic review and meta-analysis. Acta Paediatr. (2015) 104:30–7. 10.1111/apa.13133 26192560

[4] Horta BL Loret De Mola C Victora CG. Breastfeeding and intelligence: a systematic review and meta-analysis. Acta Paediatr Int J Paediatr. (2015) 104:14–9. 10.1111/apa.13139 26211556

[5] Infant and Young Child Feeding. Available online at: https://www.who.int/news-room/fact-sheets/detail/infant-and-young-child-feeding (accessed April 1, 2022).

[6] Ministry Ministry of Health Community Development Gender E and C (MoHCDGEC) [Tanzania Mainland] Ministry Ministry of Health (MoH) [Zanzibar] National National Bureau of Statistics (NBS) O of the C Government Statistician (OCGS) and I. Tanzania. Tanzania Demogr Heal Surv Malar Indic Surv 2015-16 Dar es Salaam, Tanzania, Rockville, Maryland, USA MoHCDGEC, MoH, NBS, OCGS, ICF. (2016).

[7] Kazaura M. Exclusive breastfeeding practices in the coast region, Tanzania. Afr Health Sci. (2016) 16:44–50. 10.4314/ahs.v16i1.6 27358612 PMC4915437

[8] Chandhiok N Singh KJ Sahu D Singh L Pandey A. Changes in exclusive breastfeeding practices and its determinants in India, 1992–2006: analysis of national survey data. Int Breastfeed J. (2015) 10:1–13. 10.1186/s13006-015-0059-0 26719758 PMC4696149

[9] Yalçin SS Berde AS Yalçin S. Determinants of exclusive breast feeding in Sub-saharan Africa: a multilevel approach. Paediatr Perinat Epidemiol. (2016) 30:439–49. 10.1111/ppe.12305 27259184

[10] Khanal V Sauer K Zhao Y. Exclusive breastfeeding practices in relation to social and health determinants : a comparison of the 2006 and 2011 Nepal demographic and health surveys. BMC Public Health. (2013) 13:13. 10.1186/1471-2458-13-958 24125095 PMC3852862

[11] Agho KE Dibley MJ Odiase JI Ogbonmwan SM. Determinants of exclusive breastfeeding in Nigeria. BMC Pregnancy Childbirth. (2011) 11:2–9. 10.1186/1471-2393-11-2 21219659 PMC3025918

[12] Hussein HT. Exclusive breastfeeding up to six months is very rare in tanzania: a cohort study of infant feeding practices in Kilimanjaro Area. Sci J Public Health. (2015) 3:251. 10.11648/j.sjph.20150302.24

[13] Mgongo M Hashim TH Uriyo JG Damian DJ. Stray-pedersen B, Msuya SE, et al. Determinants of exclusive breastfeeding in Kilimanjaro region, Tanzania. Sci J Public Health. (2014) 2:631–5. Available online at: http://www.sciencepublishinggroup.com/j/sjph

[14] Venancio SI Monteiro CA. Individual and contextual determinants of exclusive breast-feeding in São Paulo, Brazil: a multilevel analysis. Public Health Nutr. (2006) 9:40–6. 10.1079/PHN2005760 16480532

[15] Matanda DJ Mittelmark MB Kigaru DMD. Breast-, complementary and bottle-feeding practices in Kenya: stagnant trends were experienced from 1998 to 2009. Nutr Res. (2014) 34:507–17. 10.1016/j.nutres.2014.05.004 25026918

[16] Husain Rasheed M Philemon R Damas Kinabo G Maxym M Mamuu Shayo A Theophil Mmbaga B. Adherence to exclusive breastfeeding and associated factors in mothers of HIV-exposed infants receiving care at Kilimanjaro Christian medical centre, Tanzania. East African Heal Res J. (2018) 2:33–42. 10.24248/eahrj.v2i1.565 34308173 PMC8279206

[17] Dede KS Bras H. Exclusive Breastfeeding Patterns in Tanzania: Do Individual, Household, or Community Factors Matter? Int Breastfeed J. 15:1–11. 10.1186/s13006-020-00279-8 32321557 PMC7178598

[18] Austin PC Merlo J. Intermediate and advanced topics in multilevel logistic regression analysis. Stat Med. (2017) 36:3257–77. 10.1002/sim.7336 28543517 PMC5575471

[19] Vittinghoff E Glidden D V., Shiboski SC, McCulloch CE. Regression methods in biostatistics. Int J Neural Syst. (2012). 23:1203003. 10.1007/978-1-4614-1353-0

[20] Croft Trevor N., Aileen M. J. Marshall, Courtney K. Allen et al. Guide to DHS Statistics. Rockville, MD: ICF (2018).

[21] Shirima R Greiner T Kylberg E Gebre-Medhin M. Exclusive breast-feeding is rarely practised in rural and urban Morogoro, Tanzania. Public Health Nutr. (2001) 4:147–54. 10.1079/PHN200057 11299086

[22] Eshton Nkala T Msuya SE. Prevalence and predictors of exclusive breastfeeding among women in Kigoma region, Western Tanzania: a community based cross-sectional study. (2011). Available online at: http://www.internationalbreastfeedingjournal.com/content/6/1/17 (accessed August 1, 2020). 10.1186/1746-4358-6-17 PMC3221641 22070861

[23] Victor R Baines SK Agho KE Dibley MJ. Determinants of breastfeeding indicators among children less than 24 months of age in Tanzania: a secondary analysis of the 2010 Tanzania demographic and health survey. Available online at: http://bmjopen.bmj.com/ (accessed August 1, 2020) 10.1136/bmjopen-2012-001529 PMC3549262 23299109

[24] Mgongo M Mosha M V Uriyo JG Msuya SE. Stray-pedersen B. Prevalence and predictors of exclusive breastfeeding among women in Kilimanjaro region, Northern Tanzania : a population based cross-sectional study. Int Breastfeed J. (2013) 8:1–8. 10.1186/1746-4358-8-12 24107593 PMC3852397

[25] Sinha B Chowdhury R Sankar MJ Martines J Taneja S Mazumder S. Interventions to improve breastfeeding outcomes: a systematic review and meta-analysis. Acta Paediatr Int J Paediatr. (2015) 104:114–35. 10.1111/apa.13127 26183031

[26] Menon P Nguyen PH Saha KK Khaled A Kennedy A Tran LM. Impacts on breastfeeding practices of at-scale strategies that combine intensive interpersonal counseling, mass media, and community mobilization: results of cluster-randomized program evaluations in Bangladesh and Viet Nam. PLoS Med. (2016) 13:1–28. 10.1371/journal.pmed.1002159 27780198 PMC5079648

[27] Ekholuenetale M Barrow A Arora A. Skin-to-skin contact and breastfeeding practices in Nigeria: a study of socioeconomic inequalities. Int Breastfeed J. (2022) 17:1–12. 10.1186/s13006-021-00444-7 34980169 PMC8725355

[28] Khanal V Lee AH Scott JA Karkee R Binns CW. Implications of methodological differences in measuring the rates of exclusive breastfeeding in Nepal: findings from literature review and cohort study. BMC Pregnancy Childbirth. (2016) 16:389. 10.1186/s12884-016-1180-9 27955620 PMC5154002

[29] WHO. “Indicators for assessing breastfeeding practices WHO/CDD/SER/91.14,” in *World Health Organization*. (1991). p. 1–14. Available online at: http://apps.who.int/iris/bitstream/10665/62134/1/WHO_CDD_SER_91.14.pdf

